# CRISPR-mediated genome editing of wheat for enhancing disease resistance

**DOI:** 10.3389/fgeed.2025.1542487

**Published:** 2025-02-25

**Authors:** Joshua Waites, V. Mohan Murali Achary, Easter D. Syombua, Sarah J. Hearne, Anindya Bandyopadhyay

**Affiliations:** Genetic Resource Program, International Maize and Wheat Improvement Center (CIMMYT), El Batan, Mexico

**Keywords:** gene editing, genome editing, wheat, CRISPR, disease resistance, NLR, *R* gene, *S* gene

## Abstract

Wheat is cultivated across diverse global environments, and its productivity is significantly impacted by various biotic stresses, most importantly but not limited to rust diseases, Fusarium head blight, wheat blast, and powdery mildew. The genetic diversity of modern cultivars has been eroded by domestication and selection, increasing their vulnerability to biotic stress due to uniformity. The rapid spread of new highly virulent and aggressive pathogen strains has exacerbated this situation. Three strategies can be used for enhancing disease resistance through genome editing: introducing resistance (*R*) gene-mediated resistance, engineering nucleotide-binding leucine-rich repeat receptors (NLRs), and manipulating susceptibility (*S*) genes to stop pathogens from exploiting these factors to support infection. Utilizing *R* gene-mediated resistance is the most common strategy for traditional breeding approaches, but the continuous evolution of pathogen effectors can eventually overcome this resistance. Moreover, modifying *S* genes can confer pleiotropic effects that hinder their use in agriculture. Enhancing disease resistance is paramount for sustainable wheat production and food security, and new tools and strategies are of great importance to the research community. The application of CRISPR-based genome editing provides promise to improve disease resistance, allowing access to a broader range of solutions beyond random mutagenesis or intraspecific variation, unlocking new ways to improve crops, and speeding up resistance breeding. Here, we first summarize the major disease resistance strategies in the context of important wheat diseases and their limitations. Next, we turn our attention to the powerful applications of genome editing technology in creating new wheat varieties against important wheat diseases.

## 1 Introduction

Wheat is a vital crop that provides up to 20% of human calorie intake; however, its production is jeopardized by future environmental stresses exacerbated by climate change. As such, maintaining yield will be crucial to meeting the escalating food security demands under a changing climate. Wheat diseases significantly reduce global production, leading to losses of 20% or more of the crop annually (Savary et al., 2019). Some major wheat diseases are rust (stripe rust, leaf rust, and stem rust), wheat blast, Fusarium head blight (FHB), powdery mildew, and other bacterial, nematode, and viral diseases. A changing climate and increased global trade can accelerate the spread and the emergence of new diseases, complicating disease resistance breeding efforts. For instance, climate models forecast conditions that will facilitate the expansion of damaging wheat diseases, which will further exacerbate the challenges to future food security (Pequeno et al., 2024). The development and deployment of wheat varieties exhibiting robust and long-lasting disease resistance are crucial for combating new and existing diseases for several reasons. First, disease resistance helps stabilize yields and allows farmers to avoid substantial economic losses from crop damage and reduced production. Second, host disease resistance can slow pathogen spread and reproduction, managing severe outbreaks. Lastly, deploying disease-resistant cultivars can reduce reliance on pesticide applications, thereby representing a more cost-effective approach for farmers as well as benefiting the environment and human health (Cowger et al., 2022).

**FIGURE 1:**
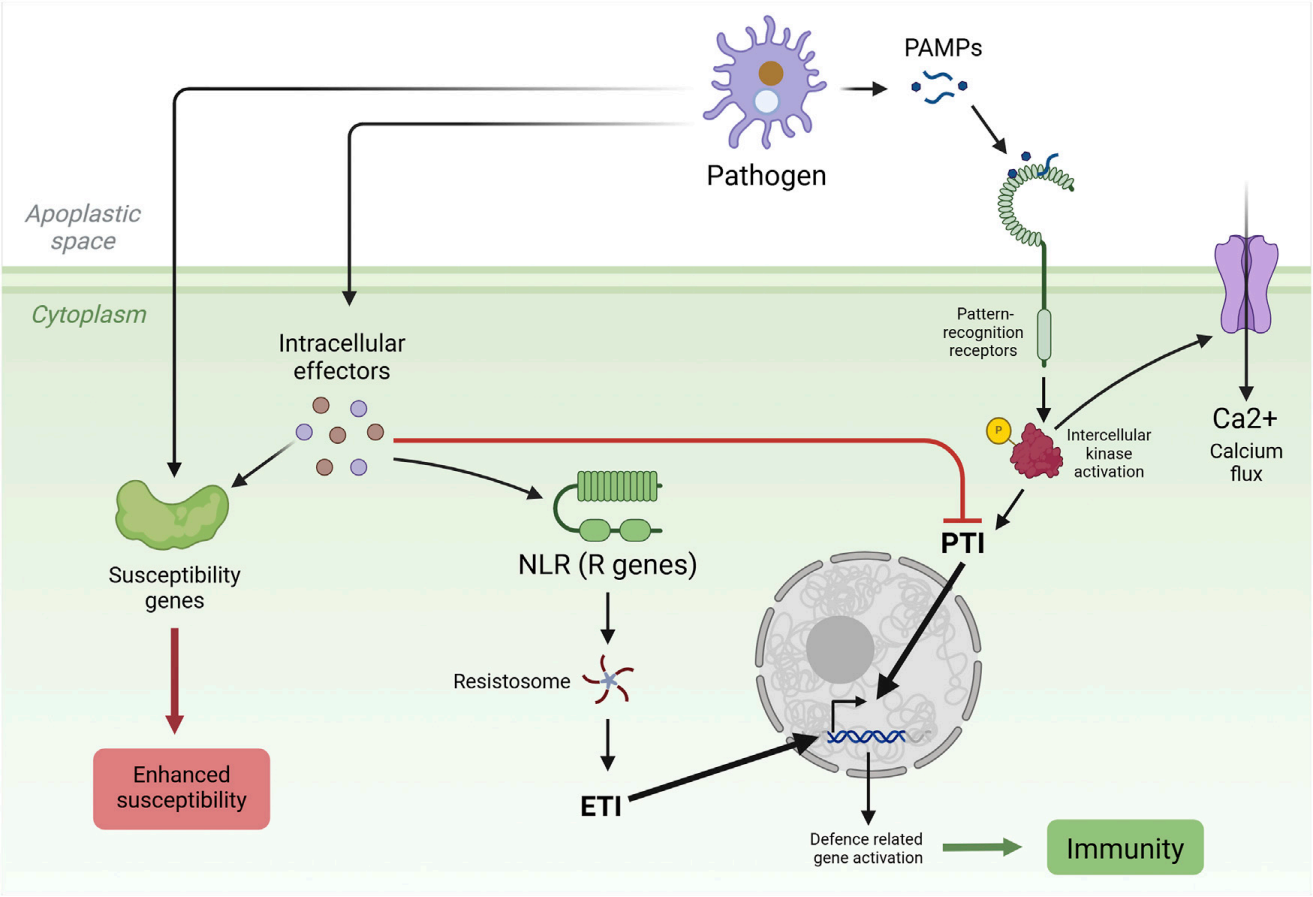
Model outlining the components and events involved in the plant immune system. The first layer of defense involves the recognition of pathogen-associated molecular patterns (PAMPs) by pattern recognition receptors (PRRs) on the cell surface, leading to pattern-triggered immunity (PTI), activation of intracellular kinases, the influx of calcium ions into the cytoplasm, the expression of defense genes, and the accumulation of antimicrobial secondary metabolites, triggering systemic acquired resistance and the hypersensitive response and overall plant immunity. The secondary immune response, known as effector-triggered immunity (ETI), is initiated upon the suppression of the PTI by pathogen-deployed effectors. Nucleotide-binding leucine-rich repeat receptors (NLRs) recognize pathogen effectors and, via resistosome complexes, trigger calcium ion influx into the plant cell, leading to ETI. Pathogens use effector proteins to counteract plant immunity by interfering with host factors encoded by susceptibility (*S*) genes, manipulating host processes, and suppressing host defense systems to establish disease. *R* gene, resistance gene. Created in https://BioRender.com.

In plants, the immune response primarily consists of two layers: pattern recognition receptors (PRRs) that act on the cell surface and nucleotide-binding leucine-rich repeat receptors (NLRs) that work in the cytoplasm. PRRs recognize pathogen-associated molecular patterns (PAMPs) in the apoplast, and NLRs identify pathogen effectors in the cytoplasm, inducing plant immune responses. PRRs activate pattern-triggered immunity (PTI) responses, and NLRs activate effector-triggered immunity (ETI) responses. Both immune responses lead to many biochemical changes inside the cell to combat infection, such as reactive oxygen species (ROS) formation, calcium burst, and hormonal reprogramming (Langner et al., 2018). PTI response leads to disease defense gene activation through various steps starting from intercellular kinase activation, and ETI response triggers numerous events downstream to effector-NLR binding, such as the involvement of helper NLRs, resistosome formation, and the induction of immune response by resistosomes (Figure 1) (Wang J. et al., 2023).

The most prominent disease resistance strategy has been introducing resistance (*R*) genes in wheat to enhance resistance against invading pathogens. *R* genes typically fall into two broad categories: all-stage resistance (ASR) and adult plant resistance (APR) genes (Norman et al., 2023). ASR genes typically provide strong, race-specific resistance throughout all growth stages, mediated by intracellular NLR immune receptors. However, they are prone to being overcome by pathogen mutations, and this is not considered a form of durable resistance. APR genes, in contrast, confer partial, race-nonspecific resistance during adult growth stages. Encoding a more diverse range of proteins, APR genes offer more durable resistance, making them highly desirable for breeders (Ellis et al., 2014). Although the process of identifying *R* genes and introgression by natural breeding is cumbersome and time-consuming, it has been an important strategy in combating plant pathogens and pests through breeding. Despite this, the selection pressures produced by deploying single *R* genes in crops promote pathogens to evolve and eventually overcome resistance conferred by these *R* genes (Langner et al., 2018). Transgenesis has been used to introduce *R* genes directly into elite material to engineer resistance against disease without introgression quickly. However, the challenge of pathogens overcoming *R* gene resistance remains the same for traditionally bred or genetically engineered disease resistance. Instead, the pyramiding of multiple *R* genes in crops has been suggested as a way to produce a selection pressure too high for the pathogen to overcome (Luo et al., 2021; Jones et al., 2024).

Pathogens exploit multiple host genes to promote successful and compatible infection. Plant genes that aid in disease progression are known as susceptibility (*S*) genes or factors. Many wheat *S* genes have been identified, and these genes help in numerous aspects of the disease progression, such as pathogen penetration, pathogen sustenance, and plant immunity suppression (Eckardt, 2002; Garcia-Ruiz et al., 2021). Inactivating *S* genes is a promising strategy for conferring disease resistance. Loss-of-function mutant generation for *S* genes through conventional mutagenesis or genome editing (GE) has enhanced disease resistance against various crop pests and pathogens. In this review, we discuss the different strategies employed for improving disease resistance in wheat, followed by detailing the application of CRISPR-mediated GE to manipulate *S* genes and *R* genes to enhance resistance. Finally, we propose new possibilities for applying an ever-increasing GE toolbox for engineering durable and robust disease resistance in wheat.

## 2 Improvement of wheat disease resistance through conventional and transgenic approaches

### 2.1 Conventional breeding approaches to improve wheat disease resistance

Conventional breeding has played a pivotal role in enhancing disease resistance in cultivated wheat, contributing significantly to protecting agricultural productivity. Conventional breeding approaches for enhancing host disease resistance involve utilizing both qualitative and quantitative resistance. Qualitative traits are controlled by major effect *R* genes, and quantitative traits are controlled by a group of minor effect genes regulated by quantitative trait loci (QTL) (Merrick et al., 2021). *R* gene-based resistance tends to be easier to use in breeding programs compared to minor gene-based resistance, which is improved gradually over multiple breeding cycles (Poland and Rutkoski, 2016). In breeding programs, *R* genes are commonly introduced into elite cultivars through marker-assisted selection; however, this can lead to linkage drag when introduced from wild or non-adapted germplasm.

As of 2021, 467 *R* genes had been designated for wheat disease resistance, with 47 of these cloned, most of which were race-specific NLRs (Hafeez et al., 2021). Moreover, for just the three rust diseases, around 920 QTLs and *R* genes have been identified, demonstrating the vast genetic resources already identified for disease-resistance breeding (Tong et al., 2024). For example, *Yr5*, *Yr10*, *Yr15,* and *Yr24/Yr26* are NLRs that provide strong race-specific resistance to stripe rust in India (Haider et al., 2023). NLRs are commonly deployed in breeding programs; however, they are prone to being overcome by pathogen evolution, especially when single *R* genes are deployed. For instance, *Sr31* provided effective stem rust resistance in wheat for 30 years before being overcome by the stem rust race Ug99 (Pretorius et al., 2000). Pyramiding multiple *R* genes is proposed as an effective strategy to prevent resistance breakdown, but conventional breeding to combine *R* genes is a very lengthy process (Hafeez et al., 2021; Wang F. et al., 2023; Jones et al., 2024). For instance, combining four broad-spectrum *R* genes for each of the three rusts (12 genes in total) would require 19 generations through a crossing approach to generate near-isogenic lines in an elite background (Hafeez et al., 2021).

### 2.2 Genetic modification approaches to improve wheat disease resistance

Integrating wheat breeding efforts and genetic engineering using *R* genes represents a sustainable approach for attaining broad-spectrum disease resistance in wheat. The introduction of cloned *R* genes through transgenesis has been demonstrated to improve the biotic resistance to many diseases in wheat; examples of this are found in Supplementary Table S1. For instance, the overexpression of the NLR *Yr10* provides robust resistance against stripe rust (Liu et al., 2014). Similarly, overexpression of the *Sr13* gene confers resistance against stripe rust and *Ug99* stem rust (Zhang W. et al., 2017). However, pathogen evolution can still overcome the introduction of single *R* genes through transgenesis. To prevent this, transgenesis allows for the introduction of multiple *R* genes in a transgene stack, allowing for the pyramiding of *R* genes without lengthy and labor-intensive crossing schemes (Hafeez et al., 2021; Jones et al., 2024). Luo et al. (2021) showed that overexpressing a multigenic construct containing the *Sr45*, *Sr55*, *Sr50*, *Sr35*, and *Sr22* genes conferred broad-spectrum rust resistance in wheat. Moreover, since the *R* genes introduced in this manner are in perfect linkage, they would be easy to deploy in breeding schemes through marker-assisted selection. However, due to limitations in the size of transgene that can be transformed into wheat, there is a limit to the number of *R* genes that can be introduced on a single *R* gene stack. Additionally, transgenes are randomly integrated into the genome, which may affect genes and genetic elements around their integration site.

Transgenic RNA interference (RNAi) approaches have also been demonstrated to improve wheat disease resistance (Supplementary Table S1). For the viruses wheat yellow mosaic virus and wheat streak virus, RNAi elements targeting a polymerase gene (*NIb8*) and a replicase gene (*NIb*), respectively, enhanced disease resistance to these two viruses (Chen et al., 2014; Tatineni et al., 2020). Constitutively expressed CRISPR/Cas nucleases have been used to target viral genomes and improve viral resistance in an approach reminiscent of RNAi. For instance, wheat dwarf virus resistance was improved in wheat through the expression of CRISPR/Cas9 and single guide RNAs (sgRNAs) targeting the DNA genome of this virus (Yuan et al., 2024). However, this resistance appears short-lived due to the rapid viral evolution that circumvents the target sites of the sgRNA (Mehta et al., 2019). Nevertheless, genetic modification approaches face cumbersome, costly regulatory processes and low societal acceptance; therefore, these examples have not been translated into commercially available crops. Instead, it is hoped that the new and precise GE technologies can open the door for improving disease resistance in wheat due to their better regulatory framework and social acceptance.

## 3 CRISPR-based precision genome editing technologies

**FIGURE 2:**
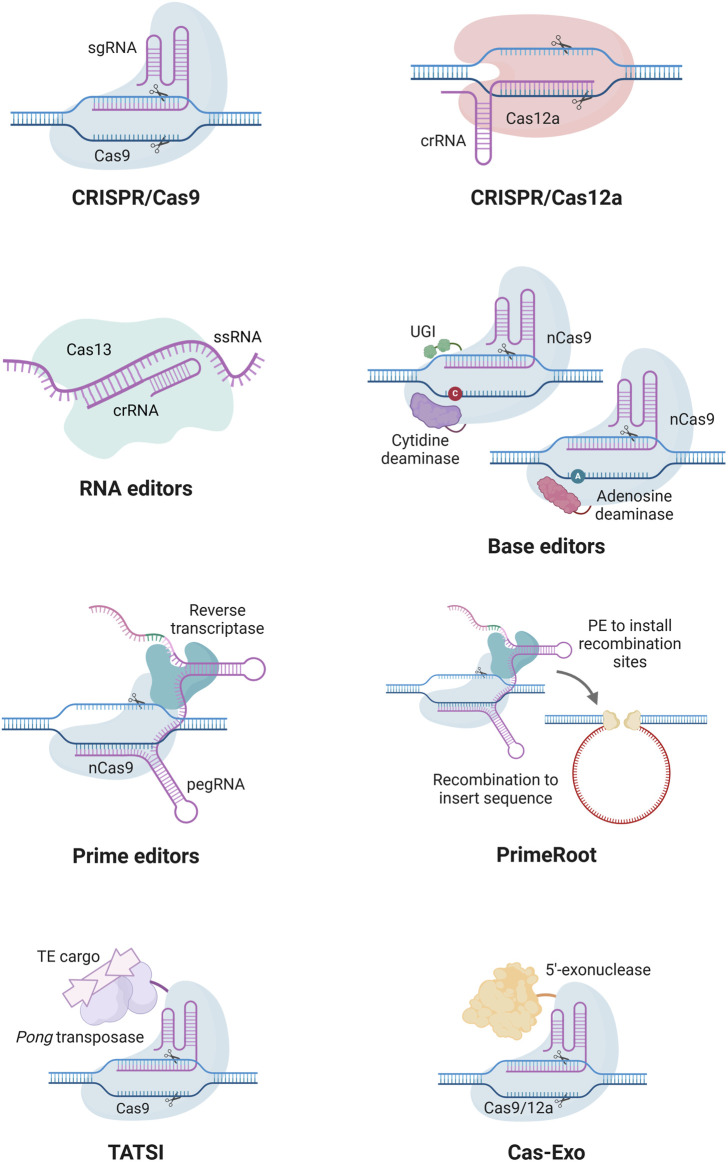
Examples of CRISPR-based tools for the modification of genomes and transcriptomes. Cas9 enables genome editing and is widely used for knockouts. Cas12a produces cohesive ends employed for both knockout, knock-ins, and easier multiplexing. Base editors integrate a nickase Cas9 (nCas9) with an adenine or cytosine deaminase to catalyze A-to-G or C-to-T substitutions, respectively. Prime editing combines nCas9 and a reverse transcriptase that uses a prime editing guide RNA (pegRNA) to install a desired edit into the target site. Cas13 is a programmable RNA-targeting system for sequence-specific recognition and cleavage. PrimeRoot utilizes a prime editor coupled with recombinases to facilitate accurate large DNA insertion. Transposase-assisted target-site integration (TATSI) utilizes a fused *Pong* transposase protein in combination with Cas9 or Cas12a to facilitate the integration of large DNA insertions. Cas-Exo harnesses Cas9 or Cas12a fused with a 5′-exonuclease to enable precise and scar-free insertion of large DNA fragments. crRNA, CRISPR RNA; ssRNA, single-stranded RNA; UGI, uracil glycosylase inhibitor; PE, prime editing; TE, transposable element. Created in https://BioRender.com.

GE enables precise modifications of DNA, including insertions, deletions, or alterations, at specific genomic loci. Several GE technologies have been developed over the years, including zinc finger nucleases (ZFNs), transcription activator-like effector nucleases (TALENs), and CRISPR/Cas nucleases (Songstad et al., 2017). While the costly and complicated protein construction associated with the protein-guided ZFNs and TALENs has limited their use, the RNA-guided CRISPR/Cas nucleases have been widely adopted due to their simplicity, cost-effectiveness, and ease of use (Figure 2). Among these, Cas9 and Cas12a nucleases are the most commonly used nucleases for GE, with engineered versions with expanded protospacer adjacent motif (PAM) sequence recognition broadening their applications in GE (Walton et al., 2020; Zeng et al., 2020; Ren et al., 2021; Liu Z. et al., 2024).

The CRISPR/*Sp*Cas9 system, derived from *Streptococcus pyogenes*, is the most widely used CRISPR system. This consists of a Cas9 nuclease and a sgRNA that directs the nuclease to a specific 20-nucleotide target site adjacent to a PAM site (Jinek et al., 2012). For *Sp*Cas9, the PAM sequence is ‘NGG’; however, CRISPR systems from other bacterial species have different PAM and target site requirements. Once bound to the target site, the Cas9 nuclease induces a double-strand break, which is subsequently repaired by endogenous cellular mechanisms. These include error-prone non-homologous end joining (NHEJ) or, less frequently, homology-directed repair (HDR) when a template sequence is present (Songstad et al., 2017). NHEJ is the primary repair pathway in plants, often introducing small insertions or deletions that can result in frameshift mutations within protein-coding sequences. While HDR is less common in plants, it enables the precise knock-ins of desired DNA sequences. The CRISPR/Cas9 technology has been widely applied to improve agronomic traits of different cereal, horticulture, and legume crops (Saini et al., 2023).

The CRISPR/Cas12a system differs from CRISPR/Cas9 by generating cohesive ends with overhangs, in contrast to the blunt ends produced by Cas9, which improve the efficiency of HDR-mediated DNA insertions (Bandyopadhyay et al., 2020). Cas12a is also a smaller protein that recognizes a ‘TTTV’ PAM sequence and cleaves DNA at a site distal to the target sequence, often resulting in larger deletions. This system has been successfully utilized in key crops, such as wheat, rice, maize, and soybean (Hu et al., 2017; Kim et al., 2017; Lee et al., 2019; Liu et al., 2020). Beyond Cas9 and Cas12a, new Cas variants and ultra-compact Cas nucleases are further expanding GE possibilities (Teng et al., 2018; Harrington et al., 2020; Pausch et al., 2020). Moreover, RNA editing by CRISPR systems opens a new window in disease research, especially for targeting RNA viruses. Cas13 and its variants are considered one of the emerging tools for diverse RNA targeting in plants, such as plant-adapted virus resistance, RNA knockdown, alternative splicing modulation, and RNA base editing (Mahas et al., 2019).

Expanding on the precision of CRISPR systems, base editing enables single-base transition substitutions without inducing DNA double-strand breaks or requiring donor templates (Komor et al., 2016). The two main types of base editors are cytosine base editors, which convert C:G to T:A, and adenine base editors, which convert A:T to G:C. These tools have been widely used in plant GE to achieve targeted base modifications (Molla et al., 2021). However, base editors face several limitations, including their ability to install only transition substitutions, strict sequence suitability and PAM availability requirements, and relatively low editing efficiencies.

Prime editing represents a significant advancement in GE, enabling all 12 types of base substitutions, as well as small DNA insertions and deletions, in a very precise manner (Anzalone et al., 2019). Unlike base editors, prime editors are not constrained by PAM sequence availability or specific sequence requirements, though they are generally associated with lower editing efficiencies (Hillary and Ceasar, 2022). This system relies on a nickase Cas9 (nCas9) fused with a reverse transcriptase and guided by a prime editing guide RNA (pegRNA). The pegRNA contains both a sgRNA for target recognition and a reverse transcription template encoding the desired edit. Upon binding, the nCas9 induces a nick in the DNA, allowing the pegRNA to initiate reverse transcription, integrating the desired edit into the genome (Anzalone et al., 2019). Prime editing has been successfully demonstrated in crops such as wheat (Ni et al., 2023), rice (Lin et al., 2020; Zong et al., 2022), maize (Jiang et al., 2020; Qiao et al., 2023) and tomatoes (Vu et al., 2024). Improvements have also been made to increase the efficiency in plants, such as the use of an extra sgRNA to nick the non-edited strand (Lin et al., 2020), interfering with the mismatch repair pathway through RNAi (Liu X. et al., 2024), using improved prime editors with modifications to nCas9 or the reverse transcriptase (Li J. et al., 2022; Zong et al., 2022), and using engineered pegRNAs with improved RNA stability (Ni et al., 2023).

Recently, innovative technologies have emerged that facilitate large DNA insertion and knock-ins in plants. One such innovation is PrimeRoot, a novel tool enabling the precise integration of large DNA segments into plant genomes (Sun et al., 2024). This technique first employs prime editing to introduce a recombination site into the genome. Concurrently, a tyrosine recombinase excises two identical recombination sites on a donor vector, producing an intermediate donor with the desired DNA insert next to a corresponding recombination site. Finally, the tyrosine recombinase integrates the desired DNA insert into the newly established recombination site within the genome. Transposase-assisted target-site integration (TATSI) is another recent technology enabling targeted DNA insertion in plants by utilizing transposable elements (Liu P. et al., 2024). Here, a rice *Pong* transposase protein fused to Cas9 or Cas12a is used to precisely excise and insert DNA cargo into a desired target site within the genome. Another new technology, Cas-Exo, was shown to significantly improve the efficiency of HDR in plants through the fusion of 5′ exonucleases to Cas9 or Cas12a (Schreiber et al., 2024). After the Cas nuclease induces a double-strand break, the 5′ exonuclease creates a longer free 3′ end by degrading the 5′ sequence of the cut strands. This free 3′ end greatly improves the efficiency of HDR in repairing the double-strand break. This Cas-Exo technology has achieved stable and heritable knock-ins in wheat with a frequency of around 1%. As these DNA insertion methods continue to improve, they hold great potential for enhancing agronomic traits in wheat through the targeted insertion of large DNA sequences.

## 4 Genome editing approaches for enhancing disease resistance in wheat

GE technologies present transformative tools to target and modify specific genetic elements for the improvement and development of durable disease resistance in wheat. This section will explore the various GE approaches that can be employed for enhancing wheat disease resistance, from targeted *S* gene manipulation to sophisticated *R* gene knock-ins and NLR domain engineering.

### 4.1 Manipulation of susceptibility genes using genome editing

**FIGURE 3:**
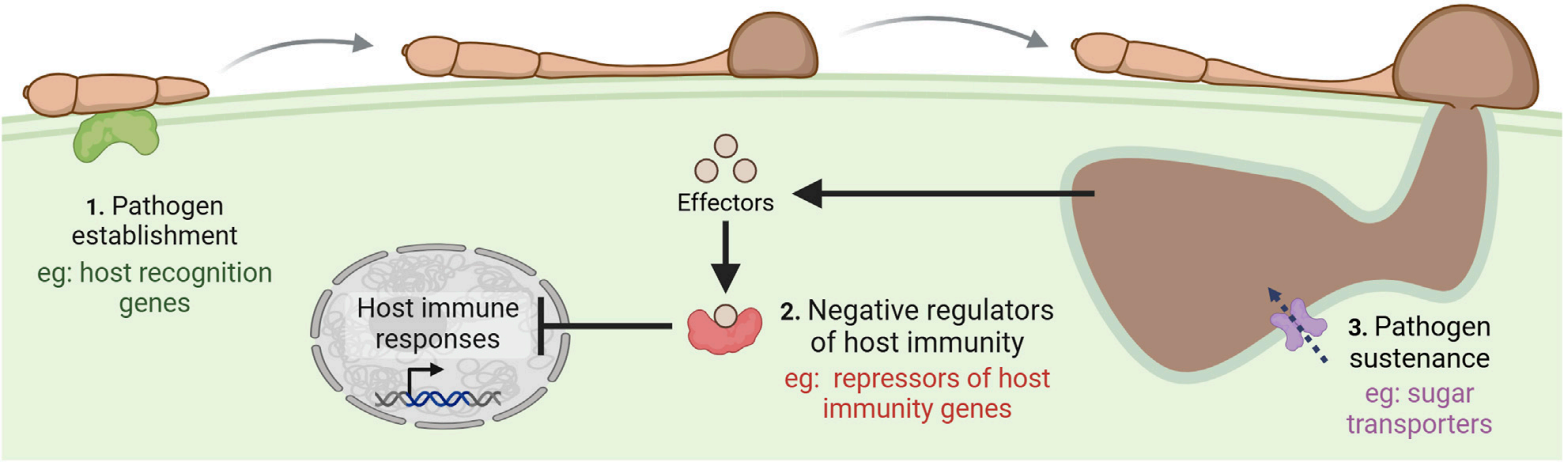
Common mechanisms of susceptibility (*S*) gene action. Pathogens employ effector molecules to target plant *S* genes, enabling them to manipulate host processes in their favor by transporting pathogen proteins within the host cell, acquiring nutrients, suppressing plant defenses, and ultimately establishing disease. Created in https://BioRender.com.

GE has been extensively demonstrated to improve wheat disease resistance through the targeted knockout of *S* genes (Figure 3). By disrupting these genes, their role in supporting compatible infection is rendered ineffective, improving the plant’s resistance to the disease. This strategy has been used to improve the biotic resistance of many crop species to viruses, bacteria, fungal pathogens, nematodes, and oomycetes (Zaidi et al., 2018; Li M. et al., 2022; Bishnoi et al., 2023). Resistance mediated by the loss of an *S* gene is thought to be more durable than *R* gene-mediated resistance since it is not based on the recognition of effectors that can rapidly evolve to overcome *R* gene-mediated resistance (Pavan et al., 2010; Gorash et al., 2021). Accordingly, examples exist of durable *S* gene-mediated resistance that have lasted for several decades in the field, demonstrating the use of this type of resistance in agriculture (Kusch and Panstruga, 2017).

Pathogens exploiting *S* genes for infection create selective pressure for their elimination, yet many *S* genes persist through plant evolution. This retention may vary by gene but is often attributed to their critical roles in host function, where loss could negatively impact plant physiology and fitness (Hückelhoven et al., 2013). For instance, *mlo*-based resistance affects agronomic traits, while *eif4e* knockouts for potyvirus resistance cause dwarfing and yield losses in several crops (Kusch and Panstruga, 2017; Hoffie et al., 2021). Despite this, there are many examples of *S* gene-mediated resistance with no associated yield penalties (Sun et al., 2016; Ingvardsen et al., 2019). It may also be possible to use GE to fine-tune the expression of essential *S* genes and uncouple the negative pleiotropic effects associated with complete loss-of-function from enhanced disease resistance. This could be done through the introduction of new untranslated open reading frames (ORFs) using base or prime editing to reduce protein accumulation (Xue et al., 2023) or through disrupting enhancer sequences within the promoter to reduce gene expression (Tang and Zhang, 2023).

Another reason that may explain their retention is that some *S* genes have been found to confer susceptibility to one pathogen while boosting resistance to another (McGrann et al., 2014). For wheat specifically, the polyploid genome and recessive nature of *S* gene-mediated resistance may make natural *S* gene loss-of-function mutations exceedingly rare due to genetic redundancy. For *mlo*-based powdery mildew resistance in wheat, the knockout of all three *TaMLO* homeologues was required to confer resistance (Wang et al., 2014). Therefore, due to this evolutionary constraint, it is possible that polyploid wheat harbors more *S* genes than diploid species, where selective pressures would more easily facilitate their elimination. This highlights the benefit of GE for the knockout of all *S* gene homeologues through multiplexing, which would otherwise be very difficult to achieve through conventional breeding methods.

The conserved function of *S* genes across plant species has facilitated their identification in wheat, with most wheat *S* genes identified as orthologues of those from other crops (Table 1) (Sun et al., 2016). For instance, *eIF4E*, an *S* gene for potyvirus infection, improved resistance when knocked out using CRISPR/Cas9 in tomatoes, potatoes, melons, barley, and wheat (Moury et al., 2020; Hoffie et al., 2021; Lucioli et al., 2022; Pechar et al., 2022; Kan et al., 2023). Moreover, wheat-specific *S* genes have been identified through cloning resistance loci, such as *TaHRC-3B*, which underlies the *Fhb1* locus conferring FHB resistance (Su et al., 2019; Chen et al., 2022). Another approach involves identifying host genes upregulated during pathogen infection and then validating their role as an *S* gene through virus-induced gene silencing (VIGS) in wheat. For example, *TaPsIPK1* expression increases during stripe rust infection, and its VIGS knockdown enhances stripe rust resistance (Wang et al., 2022b). Subsequently, its CRISPR/Cas9-mediated knockout improved resistance to stripe rust. Published databases of differentially expressed genes during pathogen infection are available, and these could be mined to identify new possible *S* genes (Ma et al., 2009). Identifying host genes that are upregulated during compatible but not incompatible infections also helps to identify possible *S* genes, and then VIGS can be used to confirm their role in susceptibility. This approach has been widely applied in wheat, especially for stripe rust and powdery mildew, identifying genes that could be future targets for knockout using GE (as reviewed in Li S. et al., 2022; Taj et al., 2022).

**TABLE 1:** Susceptibility gene examples in wheat that were targeted using genome editing tools.

Disease(s)	Gene(s)	Susceptibility (S) gene identification	Proposed susceptibility mechanism	Protein description	Pleiotropic effect	References
Powdery mildew	*TaMLO1*	Orthologue of known *S* gene	Unknown; suggested to be involved in preventing pathogen establishment or immunity suppression	Plasma membrane protein with seven-transmembrane domains	Growth and yield penalties	Wang et al. (2014)
Powdery mildew	*TaEDR1*	Orthologue of known *S* gene	Unknown; suggested to be involved in preventing pathogen establishment or immunity suppression	Raf-like mitogen-activated protein kinase kinase kinase	No obvious growth penalties	Zhang et al. (2017b)
Powdery mildew	*TaMLO1*	Orthologue of known *S* gene	Unknown; suggested to be involved in preventing pathogen establishment or immunity suppression	Plasma membrane protein with seven-transmembrane domains	No growth or yield penalties	Li et al. (2022c)
Stripe rust and Powdery mildew	*TaMKP1*	Orthologue of known *S* gene	Negative regulator of plant immune response	Mitogen-activated protein kinase phosphatase	Increased plant height, spike length, grain size and grain weight	Liu et al. (2024c)
Stripe rust	*TaWRKY19*	Orthologue of known *S* gene	Negative regulator of plant immune response	WRKY transcription factor	Authors did not mention investigating this	Wang et al. (2022a)
Stripe rust	*TaCIPK14*	Orthologue of known *S* gene/Differential gene expression during infection	Negative regulator of plant immune response	Calcineurin B-like protein-interacting protein kinase	No growth or yield penalties	He et al. (2023)
Leaf and Stripe rust	*TaPsIPK1*	Differential gene expression during infection	Effector target; suggested to negatively regulate plant immune response	Receptor-like cytoplasmic serine/threonine kinase	No growth or yield penalties	Wang et al. (2022b)
Leaf rust	*TaGW2*	Differential gene expression during infection	Negative regulator of plant immune response	E3 ubiquitin ligase	Increased grain width and grain weight with no yield penalty	Liu et al. (2024b)
Fusarium head blight	*TaHRC*	Cloned resistance (*R*) gene of wheat (Fhb1)	Unknown	Histidine-rich calcium-binding protein	Authors did not mention investigating this	Su et al. (2019)
Fusarium head blight	*TaNFXL1*	Differential gene expression during infection	Unknown	Transcription factor	Authors did not mention investigating this	Brauer et al. (2020)
Fusarium head blight	*TaHRC*	Cloned *R* gene of wheat (Fhb1)	Unknown	Histidine-rich calcium-binding protein	Authors did not mention investigating this	Chen et al. (2022)
Septoria nodorum blotch, tan spot, and spot blotch	*TaTsn1*	Cloned *R* gene of wheat	Toxin sensitivity gene targeted by ToxA to induce necrosis (effector-triggered susceptibility)	Serine/threonine protein kinase domain and nucleotide binding-leucine rich repeat (NB-LRR) domain protein	Authors did not mention investigating this	Karmacharya et al. (2023)
Septoria nodorum blotch, tan spot, and spot blotch	*TaSnn5*	Orthologue of known *S* gene	Toxin sensitivity gene targeted by Tox5 to induce necrosis (effector-triggered susceptibility)	Protein kinase and major sperm protein domain protein	Authors did not mention investigating this	Poddar et al. (2023)
Septoria nodorum blotch, tan spot, and spot blotch	*TaTsn1*	Cloned *R* gene of wheat	Toxin sensitivity gene targeted by ToxA to induce necrosis (effector-triggered susceptibility)	Serine/threonine protein kinase domain and NB-LRR domain protein	Authors did not mention investigating this	Poddar et al. (2023)
Barley yellow dwarf virus	*TaIMP-α*	Orthologue of known *S* gene	Aids in transport of the virus' 17K virulence proteins into the host nucleus	Karyopherin nuclear transport receptor	No growth or yield penalties	Wang et al. (2024)
Barley yellow dwarf virus	*TaSDN1*	Orthologue of known *S* gene	Negative regulator of plant immune response by degrading antiviral RNAi	Small RNA-degrading nuclease	No growth or yield penalties	Jin et al. (2022)
Wheat yellow mosaic virus	*TaPDIL5-1*	Orthologue of known *S* gene	Unknown; suggested to be recruited by virus to act as a cellular chaperone during infection	Endoplasmic reticulum-localized chaperone	No growth or yield penalties	Kan et al. (2022)
Wheat yellow mosaic virus	*TaeIF4E*	Orthologue of known *S* gene	Utilized by virus to initiate protein translation	Eukaryotic translation initiation factor	Increased plant height and delayed heading with no yield penalty	Kan et al. (2023)

Several wheat *S* genes have been knocked out using GE to increase resistance to various pathogens, including fungal diseases and viruses. Table 1 provides details on these examples, information on how the *S* gene was identified, and if any pleiotropic effects were observed in knockout lines.

#### 4.1.1 Powdery mildew *S* gene examples

The first *S* gene knockout in wheat using GE was *TaMLO*, enhancing resistance to powdery mildew (Wang et al., 2014). TALEN-mediated knockout of all three *TaMLO* homeologues conferred strong resistance but resulted in growth and yield penalties, restricting its use in plant breeding. Later, CRISPR/Cas9 knockouts produced a mutant with a 304-kilobase deletion in *TaMLO-B1* (*Tamlo-R32*), which maintained strong resistance while avoiding yield penalties (Zhang Y. et al., 2017). Moreover, the CRISPR/Cas9 knockout of all *TaEDR1* homeologues provided moderate resistance without growth penalties, making it suitable for breeding (Liu et al., 2024c). Similarly, the knockout of all *TaMPK1* homeologues increased powdery mildew resistance (Li S. et al., 2022). Among the *TaMPK1* homeologues, the D-genome contributed the most to susceptibility, highlighting the unequal roles that homeologues can play in susceptibility.

#### 4.1.2 Rust disease *S* gene examples

CRISPR/Cas9-mediated knockout of *TaMPK1* not only improved resistance to powdery mildew but also enhanced stripe rust resistance, another biotrophic pathogen of wheat (Liu et al., 2024c). Again, the D-genome contributed the most to stripe rust resistance. This suggests that pathogens of a similar lifestyle may exploit a similar repertoire of *S* genes, highlighting the potential *S* gene-mediated resistance has for conferring broad-spectrum resistance. Similarly, the CRISPR/Cas9-mediated knockout of *TaGW2* and *TaWRKY19* also conferred resistance to leaf and stripe rust in wheat, respectively (Wang et al., 2022a; Liu et al., 2024b). Race-nonspecific resistance, defined as resistance to multiple pathogen races, has also been achieved with wheat *S* gene knockouts. For example, *TaCIPK14* knockouts improved resistance to five stripe rust races without yield penalties under field conditions (He et al., 2023). Similarly, *TaPsIPK1* knockouts enhanced the resistance to predominant epidemic Chinese races of stripe rust in field trials with no yield penalties (Wang et al., 2022b). *TaPsIPK1* knockout also improved leaf rust resistance but had no effect on stem rust resistance due to conserved effectors between stripe rust and leaf rust, but not stem rust, that target *TaPsIPK1*. This highlights the importance of testing *S* gene knockouts against multiple pathogens to uncover broad-spectrum resistance potential.

#### 4.1.3 Fusarium head blight and other fungal diseases *S* gene examples

*Fhb1* is the most effective source of FHB resistance in wheat, providing stable and broad-spectrum resistance (Cuthbert et al., 2006). A loss-of-function mutation in *TaHRC-3B* has been identified as the key determinant of *Fhb1*-mediated resistance, classifying it as *S* gene-mediated resistance (Su et al., 2019). Consequently, the CRISPR/Cas9 knockout of *TaHRC-3B* improved FHB resistance, offering a faster alternative to backcrossing for introducing this locus into wheat varieties (Su et al., 2019; Chen et al., 2022). Similarly, the knockout of all six *TaNFXL1* gene copies, a gene associated with deoxynivalenol toxin sensitivity, also enhanced FHB resistance (Brauer et al., 2020).

Other fungal pathogens, such as Septoria nodorum blotch, tan spot, and spot blotch, produce necrotrophic effectors like ToxA, which interacts with the wheat gene *TaTsn1* to cause disease (Faris et al., 2010; McDonald et al., 2018). Loss-of-function *TaTsn1* ethyl methanesulfonate (EMS) mutants are insensitive to ToxA and show increased resistance to Septoria nodorum blotch, confirming this as an *S* gene (Faris et al., 2010). CRISPR/Cas9-mediated knockout of *TaTsn1* rendered wheat plants insensitive to ToxA, with similar resistance to these fungal pathogens expected based on EMS mutant observations (Karmacharya et al., 2023; Poddar et al., 2023). Similarly, CRISPR/Cas9 knockout of *TaSnn5*, targeted by the necrotrophic effector Tox5, produced Tox5-insensitive lines that likely also enhance resistance to Septoria nodorum blotch (Poddar et al., 2023).

#### 4.1.4 Virus *S* gene examples

Nearly half of virus resistance loci in crops are recessively inherited, highlighting the prevalence of *S* gene-mediated resistance against viruses (Kang et al., 2005). However, genetic redundancy in wheat’s polyploid genome may mask such resistance, enhancing wheat’s susceptibility to viral disease. Several virus *S* gene orthologues identified in other species have been successfully targeted in wheat using GE (Table 1). *eIF4E*, a component of the translation initiation complex targeted by potyviruses, is a well-characterized *S* gene. Its knockout enhances potyvirus resistance in various crops, including wheat, where CRISPR/Cas9-mediated knockout of *TaeIF4E* homeologues increased resistance to wheat yellow mosaic virus without yield penalties (Kan et al., 2023).

Barley, with its diploid genome, serves as a useful model for identifying orthologues of wheat virus *S* genes. For instance, loss-of-function of *HvPDIL5-1* in barley confers resistance to bymoviruses, and CRISPR/Cas9 knockout of the orthologous wheat gene *TaPDIL5-1* similarly enhances resistance to wheat yellow mosaic virus without yield penalties (Yang et al., 2014; Kan et al., 2022). Similarly, reducing *HvIMP-α* expression in barley via VIGS improves barley yellow dwarf virus resistance, and the CRISPR/Cas9 knockout of *TaIMP-α* genes in wheat enhanced resistance to this virus with no negative agronomic penalties (Wang et al., 2024). Additionally, the *HvSDN1* gene supports barley yellow dwarf infection in barley, and the CRISPR/Cas9-mediated knockout of *TaSDN1* also improved resistance to this virus in wheat (Jin et al., 2022). These studies demonstrate the value of using barley as a model to translate findings to wheat, enabling effective *S* gene identification and targeting in polyploid wheat.

### 4.2 Introduction of resistance genes using genome editing

Introducing *R* genes through genetic modification bypasses the linkage drag and lengthy backcrossing issues associated with conventional gene introgression. However, this technique is limited by transgene size restrictions and the random integration of transgenes, complicating the introduction of these into safe harbors. New knock-in technologies, such as Cas-Exo, TATSI, and PrimeRoot, allow the precise introduction of desired DNA sequences into crop genomes. As the efficiencies of these technologies improve, this will allow for the targeted insertion of *R* genes at specific safe harbor loci within the genome of elite wheat cultivars already locally adapted and bred for high yields (Greenwood et al., 2023; Jones et al., 2024). This circumvents the need for introgression through backcrossing, reducing the time needed to introduce *R* genes into elite material and abolishing linkage drag from agronomically poor breeding material. Moreover, by introducing multiple *R* genes for several diseases at a single safe harbor locus, these *R* gene stacks would be easy to manage in breeding programs through marker-assisted selection since they would stay linked at a single perfect locus.

Small polymorphisms within non-NLR proteins have been found to be responsible for improving disease resistance. For instance, for the durable APR wheat gene *Lr67/Yr46/Sr55/Pm46*, a single nucleotide polymorphism in this hexose transporter determines the difference between the susceptible and resistant alleles (Moore et al., 2015; Milne et al., 2019). Similarly, for the durable APR wheat gene *Lr34/Yr18/Sr57/Pm38*, two nucleotides differ between the susceptible and resistant alleles of this ABC transporter (Krattinger et al., 2009). Such polymorphisms responsible for improved resistance could be introduced directly into elite material using precise GE tools like base editing or prime editing.

### 4.3 Manipulation of NLR resistance genes using genome editing

**FIGURE 4:**
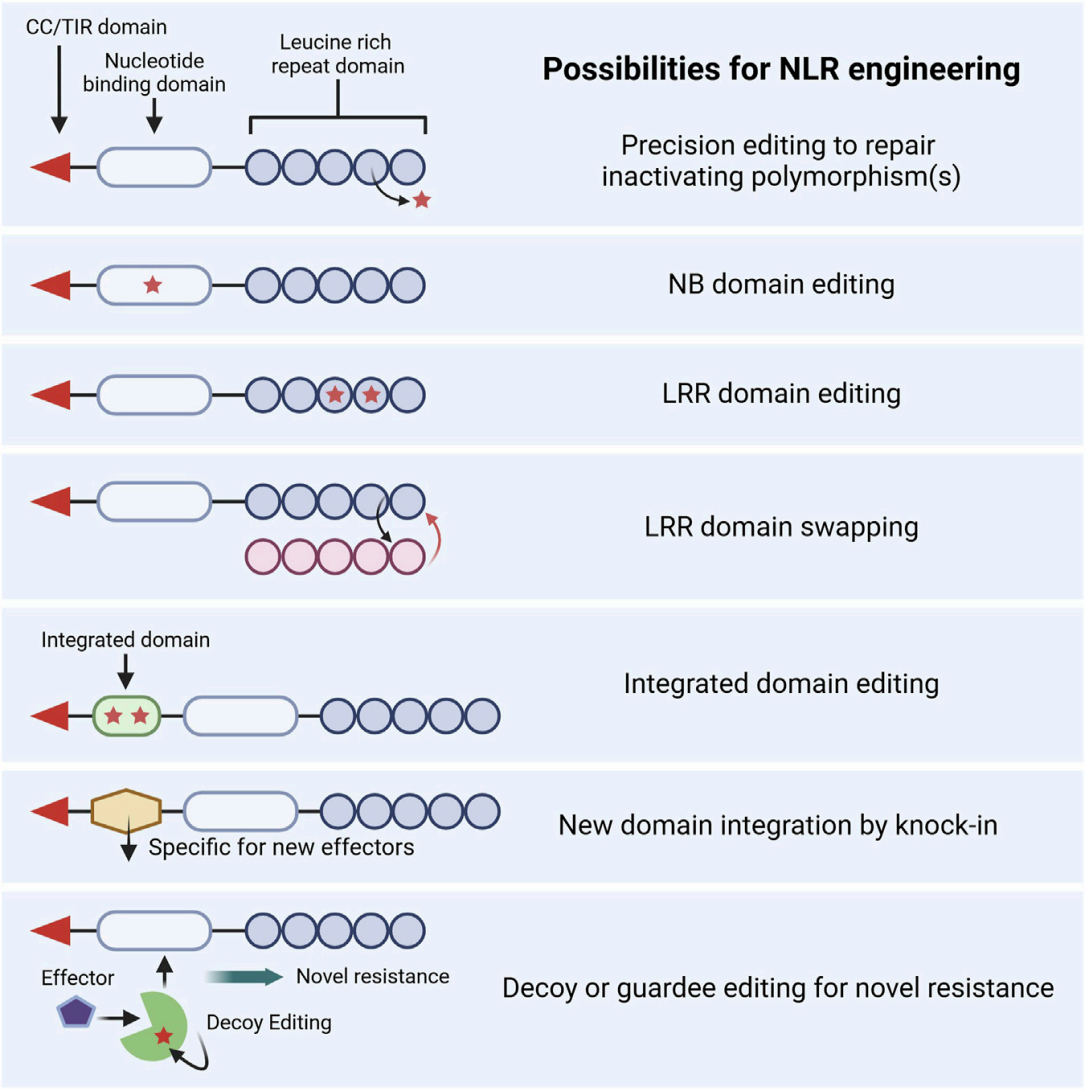
Proposed strategies for the editing of nucleotide-binding leucine-rich repeat receptors (NLRs) and associated signaling counterparts. CC, coiled-coil; TIR, toll/interleukin-1 receptor, NB, nucleotide binding; LRR, leucine-rich repeat. Created in https://BioRender.com.

The typical structure of an NLR is composed of a C-terminal coiled-coil (CC) domain or Toll/interleukin-1 receptor (TIR) enzyme domain, a central nucleotide-binding (NB) domain, and an N-terminal leucine-rich repeat (LRR) domain (Figure 4). NLRs interact either directly with their corresponding effectors or indirectly via decoy or guardee proteins. Effector interaction often occurs within their LRR domains or sometimes within integrated domains (IDs) that are located between their CC/TIR domain and NB domain or at the end of the LRR domain.

Substantial advancements in understanding the evolution, functional attributes, and structural properties of NLRs enable novel engineering strategies to improve the capacity of NLRs. NLR engineering could be used to broaden their pathogen recognition, strengthen their immune response, or create novel recognition patterns and specificities (Tamborski and Krasileva, 2020). While the efforts of CRISPR-mediated NLR engineering in wheat are limited, advancements in base editing, prime editing, and knock-in technologies open possibilities for engineering the complex domains of NLRs. This section discusses several applications of using GE to modify NLRs, as summarized in Figure 4.

#### 4.3.1 Reactivating pseudogenized NLR proteins

GE can be used to reactivate pseudogenized NLR genes and restore their functional allele directly in elite germplasm. This strategy is most suited to pseudogenized NLRs whose loss-of-function is mediated by a few polymorphisms that precise editing techniques can repair. A prior attempt to reactivate a pseudogenized NLR gene in wheat was made using GE. *lr21Ψ* differs from a functional *Lr21* allele by three nucleotide substitutions and a single base deletion that disrupts the ORF (Huang et al., 2009). TALEN-mediated editing of *lr21Ψ* in the wheat variety Fielder restored its ORF but did not reconstitute functional resistance, possibly due to consequences of the editing footprint (Luo et al., 2019). In contrast, a similar approach in rice using base editing was successful in reactivating a pseudogenized receptor-like kinase *Pi-d2*, conferring rice blast resistance (Ren et al., 2018). Through advances in the fields of comparative genomics and NLR detection, more pseudogenized wheat NLRs could be identified and reactivated similarly.

#### 4.3.2 Engineering NLR protein domains

Alterations within NB or LRR domains can broaden or modify the recognition specificities of NLRs. For instance, two polymorphisms within the NB domain of the wheat NLR Pm3 are responsible for an enhanced recognition spectrum and signaling activity of this powdery mildew *R* gene (Stirnweis et al., 2014). Similarly, the LRR domain is crucial for effector recognition specificities. For instance, the stem rust *R* genes *Sr50* and *Sr33* share a high sequence similarity but recognize different effectors (AVR-Sr50 and AVR-Sr33, respectively). Tamborski and colleagues (2023) identified the LRR domain residues responsible for the binding of Sr50 and AVR-Sr50 in wheat. They then generated a synthetic version of Sr33 containing 12 amino acid modifications derived from Sr50, which enabled the recognition of the AVR-Sr50 effector. GE technologies such as base editing, prime editing, or knock-in techniques could modify the NB or LRR domains within the endogenous NLRs of elite varieties to alter or broaden their recognition specificities. Moreover, complete LRR domain swapping using GE could modify an endogenous NLR gene to target a new set of effectors and impart resistance to a different race or disease. The feasibility of this was demonstrated in wheat and barley, where the LRR domains of TaSh1, HvSh1, HvMLA10, and HvMLA13, which do not recognize AVR-Sr35, were replaced with the LRR domain of Sr35 from wheat (Förderer et al., 2022). The co-expression of these newly engineered gain-of-function NLRs with AVR-Sr35 led to its recognition and subsequently induced cell death in *Nicotiana benthamiana* and wheat protoplasts. Moreover, HvMLA10 and HvMLA13 confer immunity in barley against barley powdery mildew effectors and share low sequence homology to Sr35, demonstrating the versatile capacity of LRR domain swapping to recognize and respond to vastly different effectors. These studies offer proof-of-concept approaches for engineering NLRs in wheat with GE.

#### 4.3.3 Engineering integrated domains

Some NLRs contain IDs that interact with pathogen effectors, mediating their recognition. These often act as effector recognition modules that mimic the host targets of effectors. GE-mediated targeted modifications in IDs or the exchange of these domains could bring about a desired change in the recognition profile of an NLR (Zdrzałek et al., 2023). Engineering of IDs has not yet been demonstrated in wheat; however, examples exist from other crops. For example, the heavy metal-associated (HMA) ID of the rice blast Pikp-1 receptor was modified based on structure-guided mutations to increase its binding with previously unrecognized AVR-Pik effector variants (De la Concepcion et al., 2018). Likewise, such domains could be modified directly within endogenous wheat NLRs using GE to change their recognition specificities.

IDs can also be exchanged for new effector recognition modules to allow for novel recognition specificities. In rice, the HMA ID of Pikp-1 was exchanged for the HMA from the host target OsHIPP19 protein to gain recognition of previously unrecognized AVR-Pik effectors (Maidment et al., 2021). Moreover, an NLR’s ID can be swapped for a different unrelated protein domain that binds a specific pathogen effector to elicit a cell death response. This was demonstrated with the replacement of the HMA ID of rice Pikp-1 with a single-domain antibody (also known as a nanobody) that recognized fluorescent proteins and triggered an immune response to plant viruses expressing these proteins (Kourelis et al., 2023). This approach may allow for the development of synthetic NLRs that recognize any desired secreted pathogen protein through the knock-in of these IDs (Zdrzałek et al., 2023).

#### 4.3.4 Engineering decoy or guardee proteins

Some NLRs recognize effectors indirectly through the detection of effector-mediated modifications to decoy or guardee proteins (Jones and Dangl, 2006; Cesari, 2018). A guardee protein is a host protein that a pathogen effector directly targets and a decoy protein is a host protein that mimics an effector target protein and exists only to enable indirect NLR detection. These guardees or decoys work as a trap for pathogen effectors monitored by NLRs that trigger immune responses after detecting effector-mediated modifications. The GE-mediated modification of guardees or decoys has been proposed as a promising approach to trap novel pathogen effectors (Veillet et al., 2020). While this has not been demonstrated within wheat, its proof-of-concept comes from *Arabidopsis*, where the cleavage of AtPBS1 by the bacterial effector AVR-PphB is monitored by the NLR receptor AtRPS5. Plants transformed with synthetic *AtPBS1* genes containing the cleavage sites of other bacterial or viral proteases resulted in the recognition of these proteases by the AtRPS5 NLR, eliciting an immune response (Kim et al., 2016). The cleavage site of endogenous AtPBS1 could be modified to include these other protease sites using GE, resulting in AtRPS5-mediated surveillance of these novel effectors (Pottinger and Innes, 2020). Similarly, the GE-mediated modification of other trap decoy or guardee proteins could confer the new ability to recognize other effectors or proteases (Veillet et al., 2020). In the future, this approach could be employed in wheat to engineer novel disease resistance.

### 4.4 Other applications of genome editing for disease resistance

GE has also been used to clone *R* genes by confirming candidate genes through GE-mediated gene knockouts, inducing susceptibility in an otherwise resistant background, thereby confirming their role in resistance. For example, CRISPR/Cas9 was used to validate the cloned *R* genes of *Yr9*, *Lr47,* and *Fhb7* in wheat (Li et al., 2023; Yu et al., 2024; Zhao et al., 2024). Cas nucleases have also been applied in pathogen diagnostics and resistance genotyping. Cas12a has enabled sensitive lateral flow assays to detect FHB in wheat grains (Mu et al., 2022; Zhang et al., 2023), and dead Cas9 has been used in lateral flow-based assays to genotype resistance alleles like *Lr34* and *Lr67* in wheat varieties and to detect stripe rust and wheat blast infections (Sánchez et al., 2022). GE also aids in pathogen characterization, with CRISPR/Cas9 utilized to identify essential FHB genes for subsequent targeting with spray-induced RNAi gene silencing methods (Kim et al., 2023). These studies demonstrate the broad applications of GE technologies for researching and improving wheat disease resistance.

## 5 Future perspectives and conclusion

The ability of CRISPR/Cas nucleases to modify multiple alleles simultaneously represents the primary benefit of this technology for polyploid wheat. This allows for generating loss-of-function mutations in all the homeologues of an *S* gene, which would otherwise be incredibly challenging to achieve through conventional breeding techniques. While recessive *S* gene-mediated resistance is prevalent in diploid species, particularly against viruses (Kang et al., 2005), it may represent an untapped source of disease resistance in wheat due to functional redundancy. To date, the main application of GE for disease resistance has been the targeted knockout of *S* genes, which has improved resistance to many of wheat’s major diseases. However, these examples have predominantly been carried out in wheat varieties amenable to transformation, such as Fielder, and the next step will be to extend this work to agronomically important varieties. Furthermore, exploring the combination of multiple *S* gene knockouts would be an important research avenue to pursue. Investigating whether such combinations can confer greater resistance to the same pathogen or resistance to multiple pathogens without inducing negative pleiotropic effects is essential for their deployment in plant breeding.

Although numerous examples of *S* gene modification using GE in wheat exist, examples of *R* gene manipulation are lacking. This scarcity likely reflects the current difficulty of achieving precise DNA editing or targeted DNA insertion in wheat. However, as prime editing and new DNA insertion techniques continue to improve in efficiency, their application in enhancing disease resistance by manipulating *R* genes will increase. A major initial target will be to use new DNA insertion techniques to knock in multiple *R* genes at a single heritable locus. This approach would circumvent the complex and time-consuming crossing schemes typically required to pyramid multiple *R* genes within breeding programs. Such a strategy would enable the deployment of multiple *R* genes at a single locus, providing durable resistance that does not segregate in subsequent generations, a very desirable prospect for the wheat breeding industry.

Precise editing and targeted DNA insertion technologies will also enable future structure-based engineering of existing NLRs to expand their recognition specificities. Achieving this requires a deep understanding of the molecular mechanisms of NLR-effector recognition in wheat. Recent work identifying key amino acids involved in the effector interactions in wheat of Sr35 and Sr50 demonstrates that such precise manipulations are now feasible (Förderer et al., 2022; Tamborski et al., 2023). Looking ahead, the development and deployment of synthetic NLRs in response to newly emerging diseases with little natural genetic resistance available could be possible through GE (Kourelis et al., 2023). An immediate application for this could be the design of novel NLRs against wheat blast, a disease that first emerged in Brazil in the 1980s and has since spread from South America to Africa and Asia (Singh et al., 2021). With little existing genetic diversity in wheat, resistant varieties rely on a single source of resistance (2NS/2AS translocation) that is being eroded by this pathogen. Moreover, climate models predict that wheat blast could reduce global wheat production by as much as 69 million tons (13%) annually by 2070 (Pequeno et al., 2024). Therefore, the ability to engineer and deploy new NLRs through GE offers significant potential in combating this expanding threat.

Since the introduction of CRISPR-mediated GE technology, it has been widely employed in wheat for trait development, notably improving disease resistance. Through GE technologies, the strategic combination of *R* and *S* gene-mediated resistance could provide durable resistance in wheat (Jones et al., 2024), and advancements in the discovery of new *S* genes and structure-guided engineering of NLRs will likely accelerate these efforts further. CRISPR-based GE is also poised to play a role in broadening resistance against continuously evolving and newly emerging pathogens and in accelerating wheat breeding efforts to improve disease resistance. However, it is important to recognize that GE is not a standalone solution for boosting disease resistance in crops. It must be intelligently integrated into existing conventional breeding strategies while aligning with the needs of the plant breeding industry. Additionally, the complex and evolving regulatory landscape surrounding GE poses challenges to its widespread adoption. Nevertheless, we hope that a shift towards product-based regulation in the coming decades will enable the broader application of GE to enhance disease resistance within our crops.
