# Supplementary material for: CRISPR-mediated genome editing of wheat for enhancing disease resistance
Source: Front Genome Ed. 2025 Feb 25;7:1542487. doi: 10.3389/fgeed.2025.1542487 (PMC11893844; doi:10.3389/fgeed.2025.1542487)
Supplement: Supplementary file 1 [file Table1.pdf]

Supplementary Materials:

Supplementary Table 1. Examples of transgenic approaches used for enhancing disease resistance in wheat.

Disease	R gene(s)	Strategy	Reference
Stripe Rust	*Yr10*	Overexpression of NLR protein	(Liu et al., 2014)
Stem Rust	*Sr13*	Overexpression of a coil-coiled NLR protein	(Zhang et al., 2017)
	Sr22b	Broad-spectrum resistance via transgenics	(Luo et al., 2022)
	Sr45, Sr55, Sr50, Sr35, Sr22	Multi-gene construct	(Luo et al., 2021)
	Sr22, Sr33, Sr35, Sr45	Multi-gene construct	(Hatta et al., 2021)
	Sr62	Tandem kinase resistance	(Yu et al., 2022)
	Sr43	Protein kinase resistance	(Yu et al., 2023)
	Sr9	NLR resistance	(Zhang et al., 2023)
	SrTA1662	Race-specific resistance	(Gaurav et al., 2022)
Leaf Rust	*Lr47*	Coil-coiled NLR gene resistance	(Li et al., 2023)
Multi-pathogen	*Lr67* (G144R variants)	Enhanced resistance via mutation	(Milne et al., 2024)
	HvWRKY6	Elicits salicylic acid signaling	(Li et al., 2022)
	Hvchi26	Barley chitinase for rust and mildew resistance	(Eissa et al., 2017)
	TaCERK1, AtCERK1	Chitin receptor-mediated resistance	(Wang et al., 2024)
Leaf Rust & Common Root Rot	*TaTLP1*	Thaumatin-like protein resistance	(Wang et al., 2020)
Powdery Mildew	*Pm17*	Transgenic overexpression	(Koller et al., 2024)
	HSP90.2	Protein folding pathway resistance	(Yan et al., 2023)
	TiAP1	Aspartic protease protein resistance	(Yang et al., 2022)
Fusarium Head Blight	*TaUGT6*	UDP-glycosyltransferase resistance	(He et al., 2020)
	TaFROG	Stabilization of TaSnRK1α	(Jiang et al., 2020)

	NbRXEG1	Heterologous expression of tobacco gene	(Wang et al., 2023)
	Fhb7	Glutathione S-transferase resistance	(Zhao et al., 2024)
	TaCAT2	Lesion mimic	(Han et al., 2024)
	TaUGT3	Resistance to DON	(Lulin et al., 2010)
	TaICSA	Enhanced salicylic acid levels	(Zhang et al., 2024)
	TaPIP2;10	Aquaporin gene resistance	(Wang et al., 2021)
Fusarium Crown Rot	*TdRCA1*	Resistance via a transcription factor	(Li et al., 2024)
Fusarium Head Blight & Crown Rot	*HvUGT13248, AcPMEI, PvPGIP2*	Transgene pyramiding	(Mandalà et al., 2021)
Sharp Eyespot	*TaSTT3b-2B, TaRCR1*	Resistance via overexpression	(Zhu et al., 2017, 2022)
Septoria Tritici Blotch	*Stb16q*	Resistance via gene overexpression	(Saintenac et al., 2021)
Chinese Wheat Mosaic Virus	*TaTHI2*	Ca^{2+}-dependent protein kinase interaction	(Yang et al., 2024)
Rice Black-Streaked Dwarf Virus	*qMrdd2*	Transcription factor ZmGLK36	(Xu et al., 2023)
Wheat Yellow Mosaic Virus	*NIb8*	Antisense virus polymerase gene	(Chen et al., 2014)
Wheat Streak Mosaic Virus & Triticum Mosaic Virus	RNAi hybrid *NIb*	RNAi-mediated dual resistance	(Tatineni et al., 2020)

References:

Chen, M., Sun, L., Wu, H., Chen, J., Ma, Y., Zhang, X., et al. (2014). Durable field resistance to wheat yellow mosaic virus in transgenic wheat containing the antisense virus polymerase gene. *Plant Biotechnology Journal* 12, 447–456. doi: 10.1111/pbi.12151

Eissa, H. F., Hassanien, S. E., Ramadan, A. M., El-Shamy, M. M., Saleh, O. M., Shokry, A. M., et al. (2017). Developing transgenic wheat to encounter rusts and powdery mildew by overexpressing barley chi26 gene for fungal resistance. *Plant Methods* 13, 41. doi: 10.1186/s13007-017-0191-5

Gaurav, K., Arora, S., Silva, P., Sánchez-Martín, J., Horsnell, R., Gao, L., et al. (2022). Population genomic analysis of Aegilops tauschii identifies targets for bread wheat improvement. *Nat Biotechnol* 40, 422–431. doi: 10.1038/s41587-021-01058-4

Han, S., Han, X., Li, Y., Li, K., Yin, J., Gong, S., et al. (2024). Wheat lesion mimic homology gene *TaCAT2* enhances plant resistance to biotic and abiotic stresses. *International Journal of Biological Macromolecules* 277, 134197. doi: 10.1016/j.ijbiomac.2024.134197

Hatta, M. A. M., Arora, S., Ghosh, S., Matny, O., Smedley, M. A., Yu, G., et al. (2021). The wheat Sr22, Sr33, Sr35 and Sr45 genes confer resistance against stem rust in barley. *Plant Biotechnology Journal* 19, 273–284. doi: 10.1111/pbi.13460

He, Y., Wu, L., Liu, X., Jiang, P., Yu, L., Qiu, J., et al. (2020). TaUGT6, a Novel UDP-Glycosyltransferase Gene Enhances the Resistance to FHB and DON Accumulation in Wheat. *Front Plant Sci* 11, 574775. doi: 10.3389/fpls.2020.574775

Jiang, C., Hei, R., Yang, Y., Zhang, S., Wang, Q., Wang, W., et al. (2020). An orphan protein of Fusarium graminearum modulates host immunity by mediating proteasomal degradation of TaSnRK1α. *Nat Commun* 11, 4382. doi: 10.1038/s41467-020-18240-y

Koller, T., Camenzind, M., Jung, E., Brunner, S., Herren, G., Armbruster, C., et al. (2024). Pyramiding of transgenic immune receptors from primary and tertiary wheat gene pools improves powdery mildew resistance in the field. *J Exp Bot* 75, 1872–1886. doi: 10.1093/jxb/erad493

Li, H., Hua, L., Zhao, S., Hao, M., Song, R., Pang, S., et al. (2023). Cloning of the wheat leaf rust resistance gene Lr47 introgressed from Aegilops speltoides. *Nat Commun* 14, 6072. doi: 10.1038/s41467-023-41833-2

Li, J., Zhang, C., Xu, X., Su, Y., Gao, Y., Yang, J., et al. (2024). A MYB family transcription factor TdRCA1 from wild emmer wheat regulates anthocyanin biosynthesis in coleoptile. *Theor Appl Genet* 137, 208. doi: 10.1007/s00122-024-04723-9

Li, M., Zhao, S., Yang, J., Ren, Y., Su, J., Zhao, J., et al. (2022). Exogenous expression of barley *HvWRKY6* in wheat improves broad-spectrum resistance to leaf rust, *Fusarium* crown rot, and sharp eyespot. *International Journal of Biological Macromolecules* 218, 1002–1012. doi: 10.1016/j.ijbiomac.2022.07.138

Liu, W., Frick, M., Huel, R., Nykiforuk, C. L., Wang, X., Gaudet, D. A., et al. (2014). The Stripe Rust Resistance Gene Yr10 Encodes an Evolutionary-Conserved and Unique CC–NBS–LRR Sequence in Wheat. *Molecular Plant* 7, 1740–1755. doi: 10.1093/mp/ssu112

Lulin, M., Yi, S., Aizhong, C., Zengjun, Q., Liping, X., Peidu, C., et al. (2010). Molecular cloning and characterization of an up-regulated UDP-glucosyltransferase gene induced by DON from Triticum aestivum L. cv. Wangshuibai. *Mol Biol Rep* 37, 785–795. doi: 10.1007/s11033-009-9606-3

Luo, J., Rouse, M. N., Hua, L., Li, H., Li, B., Li, T., et al. (2022). Identification and characterization of Sr22b, a new allele of the wheat stem rust resistance gene Sr22 effective against the Ug99 race group. *Plant Biotechnology Journal* 20, 554–563. doi: 10.1111/pbi.13737

Luo, M., Xie, L., Chakraborty, S., Wang, A., Matny, O., Jugovich, M., et al. (2021). A five-transgene cassette confers broad-spectrum resistance to a fungal rust pathogen in wheat. *Nat Biotechnol* 39, 561–566. doi: 10.1038/s41587-020-00770-x

Mandalà, G., Ceoloni, C., Busato, I., Favaron, F., and Tundo, S. (2021). Transgene pyramiding in wheat: Combination of deoxynivalenol detoxification with inhibition of cell wall degrading enzymes to contrast Fusarium Head Blight and Crown Rot. *Plant Science* 313, 111059. doi: 10.1016/j.plantsci.2021.111059

Milne, R. J., Dibley, K. E., Bose, J., Riaz, A., Zhang, J., Schnippenkoetter, W., et al. (2024). Dissecting the causal polymorphism of the *Lr67res* multipathogen resistance gene. *Journal of Experimental Botany*, erae164. doi: 10.1093/jxb/erae164

Saintenac, C., Cambon, F., Aouini, L., Verstappen, E., Ghaffary, S. M. T., Poucet, T., et al. (2021). A wheat cysteine-rich receptor-like kinase confers broad-spectrum resistance against Septoria tritici blotch. *Nat Commun* 12, 433. doi: 10.1038/s41467-020-20685-0

Tatineni, S., Sato, S., Nersesian, N., Alexander, J., Quach, T., Graybosch, R. A., et al. (2020). Transgenic Wheat Harboring an RNAi Element Confers Dual Resistance Against Synergistically Interacting Wheat Streak Mosaic Virus and Triticum Mosaic Virus. *MPMI* 33, 108–122. doi: 10.1094/MPMI-10-19-0275-R

Wang, F., Yuan, S., Wu, W., Yang, Y., Cui, Z., Wang, H., et al. (2020). TaTLP1 interacts with TaPR1 to contribute to wheat defense responses to leaf rust fungus. *PLoS Genet* 16, e1008713. doi: 10.1371/journal.pgen.1008713

Wang, L., He, Y., Guo, G., Xia, X., Dong, Y., Zhang, Y., et al. (2024). Overexpression of plant chitin receptors in wheat confers broad-spectrum resistance to fungal diseases. *The Plant Journal* 120, 1047–1063. doi: 10.1111/tpj.17035

Wang, X., Lu, K., Yao, X., Zhang, L., Wang, F., Wu, D., et al. (2021). The Aquaporin TaPIP2;10 Confers Resistance to Two Fungal Diseases in Wheat. *Phytopathology* 111, 2317–2331. doi: 10.1094/PHYTO-02-21-0048-R

Wang, Z., Yang, B., Zheng, W., Wang, L., Cai, X., Yang, J., et al. (2023). Recognition of glycoside hydrolase 12 proteins by the immune receptor RXEG1 confers Fusarium head blight resistance in wheat. *Plant Biotechnology Journal* 21, 769–781. doi: 10.1111/pbi.13995

Xu, Z., Zhou, Z., Cheng, Z., Zhou, Y., Wang, F., Li, M., et al. (2023). A transcription factor ZmGLK36 confers broad resistance to maize rough dwarf disease in cereal crops. *Nat. Plants* 9, 1720–1733. doi: 10.1038/s41477-023-01514-w

Yan, Y., Guo, Y.-T., Chang, C.-Y., Li, X.-M., Zhang, M.-Q., Ding, C.-H., et al. (2023). HSP90.2 modulates 2Q2-mediated wheat resistance against powdery mildew. *Plant, Cell & Environment* 46, 1935–1945. doi: 10.1111/pce.14579

Yang, J., Chen, L., Zhang, J., Liu, P., Chen, M., Chen, Z., et al. (2024). TaTHI2 interacts with Ca2+-dependent protein kinase TaCPK5 to suppress virus infection by regulating ROS accumulation. *Plant Biotechnology Journal* 22, 1335–1351. doi: 10.1111/pbi.14270

Yang, Y., Fan, P., Liu, J., Xie, W., Liu, N., Niu, Z., et al. (2022). Thinopyrum intermedium TiAP1 interacts with a chitin deacetylase from Blumeria graminis f. sp. tritici and increases the resistance to Bgt in wheat. *Plant Biotechnology Journal* 20, 454–467. doi: 10.1111/pbi.13728

Yu, G., Matny, O., Champouret, N., Steuernagel, B., Moscou, M. J., Hernández-Pinzón, I., et al. (2022). Aegilops sharonensis genome-assisted identification of stem rust resistance gene Sr62. *Nat Commun* 13, 1607. doi: 10.1038/s41467-022-29132-8

Yu, G., Matny, O., Gourdoupis, S., Rayapuram, N., Aljedaani, F. R., Wang, Y. L., et al. (2023). The wheat stem rust resistance gene Sr43 encodes an unusual protein kinase. *Nat Genet* 55, 921–926. doi: 10.1038/s41588-023-01402-1

Zhang, J., Nirmala, J., Chen, S., Jost, M., Steuernagel, B., Karafiatova, M., et al. (2023). Single amino acid change alters specificity of the multi-allelic wheat stem rust resistance locus SR9. *Nat Commun* 14, 7354. doi: 10.1038/s41467-023-42747-9

Zhang, W., Chen, S., Abate, Z., Nirmala, J., Rouse, M. N., and Dubcovsky, J. (2017). Identification and characterization of Sr13, a tetraploid wheat gene that confers resistance to the Ug99 stem rust race group. *Proceedings of the National Academy of Sciences* 114, E9483–E9492. doi: 10.1073/pnas.1706277114

Zhang, Y.-Z., Man, J., Xu, D., Wen, L., Li, Y., Deng, M., et al. (2024). Investigating the mechanisms of isochorismate synthase: An approach to improve salicylic acid synthesis and increase resistance to Fusarium head blight in wheat. *The Crop Journal* 12, 1054–1063. doi: 10.1016/j.cj.2024.05.012

Zhao, L., Bernardo, A., Kong, F., Zhao, W., Dong, Y., Lee, H., et al. (2024). A Glutathione S-Transferase from Thinopyrum ponticum Confers Fhb7 Resistance to Fusarium Head Blight in Wheat. *Phytopathology®* 114, 1458–1461. doi: 10.1094/PHYTO-03-24-0106-SC

Zhu, X., Lu, C., Du, L., Ye, X., Liu, X., Coules, A., et al. (2017). The wheat NB-LRR gene Ta1 is required for host defence response to the necrotrophic fungal pathogen Rhizoctonia cerealis. *Plant Biotechnology Journal* 15, 674–687. doi: 10.1111/pbi.12665

Zhu, X., Rong, W., Wang, K., Guo, W., Zhou, M., Wu, J., et al. (2022). Overexpression of TaSTT3b-2B improves resistance to sharp eyespot and increases grain weight in wheat. *Plant Biotechnology Journal* 20, 777–793. doi: 10.1111/pbi.13760
